# Tissue-specific expression profiles and positive selection analysis in the tree swallow (*Tachycineta bicolor*) using a *de novo* transcriptome assembly

**DOI:** 10.1038/s41598-019-52312-4

**Published:** 2019-11-01

**Authors:** Alexandra B. Bentz, Gregg W. C. Thomas, Douglas B. Rusch, Kimberly A. Rosvall

**Affiliations:** 1 Department of Biology, Indiana University, Bloomington, IN 47405 USA; 2 Center for the Integrative Study of Animal Behavior, Indiana University, Bloomington, IN 47405 USA; 3 Department of Computer Science, Indiana University, Bloomington, IN 47405 USA; 4 Center for Genomics and Bioinformatics, Indiana University, Bloomington, IN 47405 USA

**Keywords:** Molecular evolution, Transcriptomics

## Abstract

Tree swallows (*Tachycineta bicolor*) are one of the most commonly studied wild birds in North America. They have advanced numerous research areas, including life history, physiology, and organismal responses to global change; however, transcriptomic resources are scarce. To further advance the utility of this system for biologists across disciplines, we generated a transcriptome for the tree swallow using six tissues (brain, blood, ovary, spleen, liver, and muscle) collected from breeding females. We *de novo* assembled 207,739 transcripts, which we aligned to 14,717 high confidence protein-coding genes. We then characterized each tissue with regard to its unique genes and processes and applied this transcriptome to two fundamental questions in evolutionary biology and endocrinology. First, we analyzed 3,015 single-copy orthologs and identified 46 genes under positive selection in the tree swallow lineage, including those with putative links to adaptations in this species. Second, we analyzed tissue-specific expression patterns of genes involved in sex steroidogenesis and processing. Enzymes capable of synthesizing these behaviorally relevant hormones were largely limited to the ovary, whereas steroid binding genes were found in nearly all other tissues, highlighting the potential for local regulation of sex steroid-mediated traits. These analyses provide new insights into potential sources of phenotypic variation in a free-living female bird and advance our understanding of fundamental questions in evolutionary and organismal biology.

## Introduction

The rapid increase of available transcriptomes for non-model organisms over the past few years has greatly contributed to ecological and evolutionary advances in natural systems^1–3^. This is particularly true for avian species^4^, yet one key species used in eco-evolutionary and organismal biology has a surprising absence of molecular resources. Tree swallows (*Tachycineta bicolor*) are one of the best-studied, free-living native species in North America^5^. They are found continent-wide and their willingness to breed in artificial nest boxes makes them easy to observe and experimentally manipulate. This has led to decades of data on their breeding ecology and life-history, which has advanced understanding of the effects of anthropogenic global change^6^, ecotoxicology^7^, and trade-offs in physiology and behavior^8–10^. Tree swallows are arguably the “white rat” of free-living, North American birds^11^, yet only a handful of studies have performed candidate gene analyses^12–16^. A reference genome was recently assembled for this species^17^, but a genome lacks the ability to indicate gene function, whereas transcriptomic data can facilitate a better understanding of the molecular underpinnings of phenotypic plasticity. Thus, while tree swallows are an invaluable free-living model organism for exploring phenotypic responses to social and environmental variation, transcriptomic resources are sorely needed. Moreover, the wealth of prior research in this system makes the tree swallow well-suited to address fundamental questions in evolutionary and organismal biology.

A major goal in evolutionary biology is to identify genes that are subject to adaptive evolution (i.e., evolving under positive selection)^18^. Avian transcriptomes have been assembled for a wide range of species^4^, which holds promise for new insights into the molecular processes driving adaptation. For example, research in mammals suggests that genes expressed at low levels and those that are tissue‐specific may evolve more rapidly^19^, and work thus far in birds largely mirrors these findings^20–22^. However, only a few studies have used comparative transcriptomics to investigate evolutionary patterns between bird lineages^23–26^, and even fewer include multiple independent tissues^22^. Tree swallows are well-suited for exploring the relationship between protein evolution and tissue specificity, particularly related to tissues involved in reproductive competition and immune function. As aerial insectivores that feed on emergent aquatic insects, they are more susceptible to accumulated contaminants^27^ and this can negatively impact immune function^28^ and reproductive success^29^. Tree swallows are also secondary-cavity nesters with limited nesting sites, which generates steep competition for territories and a substantive population of non-breeding individuals^30^. Predictably, tree swallows have one of the highest rates of extra-pair paternity of any socially monogamous songbird (50–90% of nests contain extra-pair young)^31–33^. Thus, this system can facilitate the discovery of protein-coding genes undergoing positive selection, providing insights into lineage-specific adaptations related to competition and immunity.

Transcriptomes can also be used to explore candidate gene pathways to improve understanding of organismal biology^34,35^. Sex steroids, such as testosterone and estradiol, regulate many physiological and behavioral traits, and there is growing interest in whether or how different sex steroid-mediated traits can evolve in concert or independently^36^. Specifically in females, selection is hypothesized to favor mechanisms that allow for greater phenotypic independence^37^, where traits are less dependent on the direct actions of circulating steroids due to tissue-specific variation in steroid production and/or sensitivity. Unfortunately, most prior work in this field of study has only focused on genes that function downstream of androgen production (e.g., androgen and estrogen receptors and aromatase) in a limited set of tissues (e.g., gonad and brain)^38^. Female tree swallows are especially amenable for addressing these knowledge gaps because they fiercely compete to obtain^9^ and maintain^39,40^ limited nesting cavities and this aggression is, in part, mediated by sex steroids^41^, the consequences of which have important transgenerational effects^42,43^. Future work providing detailed knowledge of tissue-specific regulation of the full suite of steroid processing genes would greatly improve our understanding of the potential for correlated phenotypic evolution across tissues.

Here, we present a functionally annotated tree swallow transcriptome generated from six tissues (brain, blood, ovary, muscle, spleen, and liver) in two females. To capture genes expressed across the breeding season, one female was collected early in the breeding season during territory establishment and another was collected later during incubation. Because we included multiple tissues, we also identified unique genes and processes to better characterize tissue functions. In addition, we report on two initial applications of this new molecular resource. First, to identify genes that are more rapidly evolving in the tree swallow lineage, we performed a comparative analysis of protein-coding sequences, comparing our transcriptome to 8 other avian species with high-quality sequence data. We further analyze characteristics of positively selected genes thought to impact protein evolution^19^, including gene expression levels and tissue specificity, with a focus on the tissue-specific functions identified here. We relate these findings back to tree swallow life history for a greater understanding of molecular evolution in this exceptionally well-studied wild bird. Second, we leveraged analyses of tissue-specific gene expression profiles to ask a fundamental question about organismal regulation of sex steroid-mediated phenotypes. Collectively, these analyses shed light on important questions in evolutionary biology and endocrinology, showcasing this transcriptome as a molecular tool to expand understanding of evolutionary and organismal biology.

## Results

### Assembly evaluation

Sequencing was performed with both NextSeq and MiSeq platforms to generate both short and long reads. The average number of paired-end reads per sample was ~136 million (Table S1). Trimmed reads from all six tissues were assembled into 207,739 transcripts, totaling 278,915,174 nucleotides with a mean length of 1,343 base pairs (bp) and an N50 of 1889 bp (see Table 1 for a summary of assessment metrics). Sequence length distribution of these transcripts and other assembly metrics were comparable to other avian species (Supplementary Fig. S1, Table S2). Ultimately, there were 144,119 (69.4%) transcripts that aligned to known proteins in the NCBI database using BLAST (e < 1e-10). We applied a stringent filter to remove any transcripts that did not display high confidence (with at least 50% coverage of the full-length protein and at least 70% identity), which yielded 22,825 transcripts (Table 1). Putatively unspliced introns and largely redundant transcripts were also removed resulting in a set of high confidence protein-coding segments (n = 14,717). 9,344 (63.5%) of these had alignments covering ≥ 90% of the full-length protein. Some of the annotated transcripts were identified as ‘uncharacterized’ proteins (467; 3.2%); however, we included these in subsequent analyses as some could represent unique genes to birds with unknown functions. Over 50% of annotated transcripts aligned best to *Parus major* or *Sturnus vulgaris* (Supplementary Fig. S2), and the 14,717 protein-coding genes we identified is comparable to the 15,183 in the *Parus major* assembly (Parus_major1.0.3).

**Table 1:** Summary statistics for the *de novo* assembly.

Category	Value
Total transcripts	207,739
GC (%)	45.87
**All transcript contigs**	
N50 transcript length (bp)	1,889
median contig length (bp)	809
mean contig length (bp)	1,343
total assembled bases	278,915,174
**Annotation**	
Transcripts with BLAST hits	144,119 (69.4%)
Transcripts with ≥70% identity and ≥50% coverage	22,825 (11.0%)
Genes with unique BLAST match	14,717 (7.1%)

**Figure 1:**
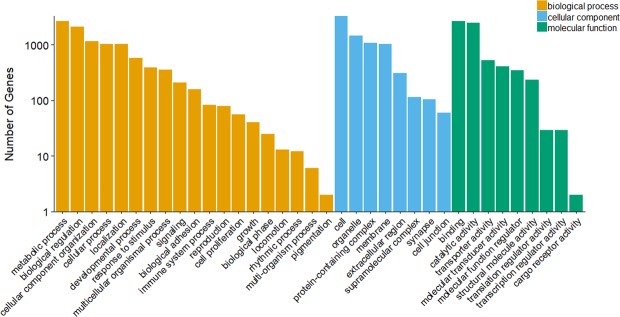
Functional classification of the *de novo* assembly of the tree swallow transcriptome. All tissues are combined and the three main Gene Ontology categories are depicted (biological process, molecular function, and cellular component).

We evaluated transcriptome assembly quality and accuracy using TransRate^44^ (including backmapping), and completeness using BUSCO (Benchmarking Universal Single-Copy Orthologs)^45^. The final transcriptome had a TransRate score of 0.38 (optimized score of 0.51) and, of the total number of assembled reads, 82% successfully mapped back to the transcriptome. Assessment of completeness using the BUSCO^45^ database of 4,915 orthologs shared among all metazoans suggested that our assembly is 91.7% complete (3,626 complete single-copy BUSCOs and 879 complete duplicated BUSCOs), with 4.8% of contigs fragmented (237 BUSCOs) and 3.5% missing (173 BUSCOs). We assigned Gene Ontology (GO) terms to 9,563 (65.0%) transcripts, and categories were well distributed among biological processes, molecular functions, and cellular components (Fig. 1). Thus, this first draft transcriptome assembly is a good foundation for future research.

### Tissue expression profiles

**Figure 2:**
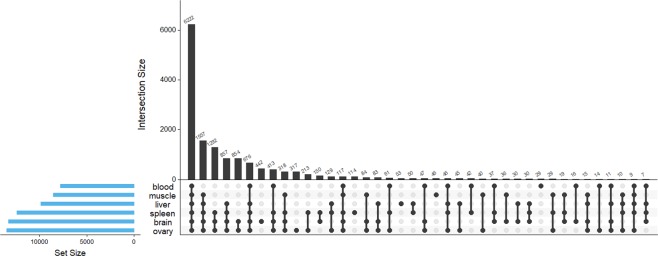
UpSet plot depicting the number of unique and shared transcripts with protein hits found in each tissue. Intersection size is the number of transcripts with TPM ≥ 1 in designated sets or groups.

An UpSet plot was constructed to compare presence/absence of genes across tissues (Fig. 2). Most of the 14,717 putative genes were expressed in the ovary (n = 13,479 genes; 91.6%), followed by brain (n = 13,332; 90.6%), spleen (n = 12,419; 84.4%), liver (n = 9,868; 67.1%), muscle (n = 8,545; 58.1%), and blood (n = 7,789; 52.9%). Libraries were constructed with equal amounts of cDNA, suggesting these differences likely reflect biological variation. We identified genes only present in one tissue to gain insight into tissue-specific functions and provide context for downstream analyses on tissue specificity and positive selection. Brain and then ovary had the most unique genes expressed, while blood had the least (Fig. 2). The most abundant, unique gene in each tissue was myelin proteolipid protein (PLP1) in brain, zona pellucida sperm-binding protein (ZP2) in ovary, C-X-C motif chemokine 2-like (CXCL2) in spleen, complement component C6 (C6) in liver, troponin C, skeletal muscle (TNNC2) in muscle, and translocator protein 2 (TSPO2) in blood. The top 10 most abundant, unique genes for each tissue are listed in Supplementary Table S3. GO overrepresentation analyses of all uniquely expressed genes for each tissue indicated that immune response (GO:0006955) was the most significant biological process in spleen, musculoskeletal movement (GO:0050881) in muscle, gene silencing by RNA (GO:0031047) in ovary, and developmental induction (GO:0031128) in brain; for a full list see Supplementary Tables S4-7. Uniquely expressed genes in liver and blood did not have overrepresented GO terms.

The greatest overlap in presence/absence of genes occurred between brain and ovary (n = 854 genes; Fig. 2), and an overrepresentation analysis indicated that these genes are involved in the regulation of hormone secretion (GO:00468; FDR = <0.001) and aggressive behavior (GO:0002118; FDR = 0.03). Spleen overlapped in expression most with ovary (n = 213 genes), liver with spleen (n = 50), blood with spleen (n = 42), and muscle with ovary (n = 40).

**Figure 3:**
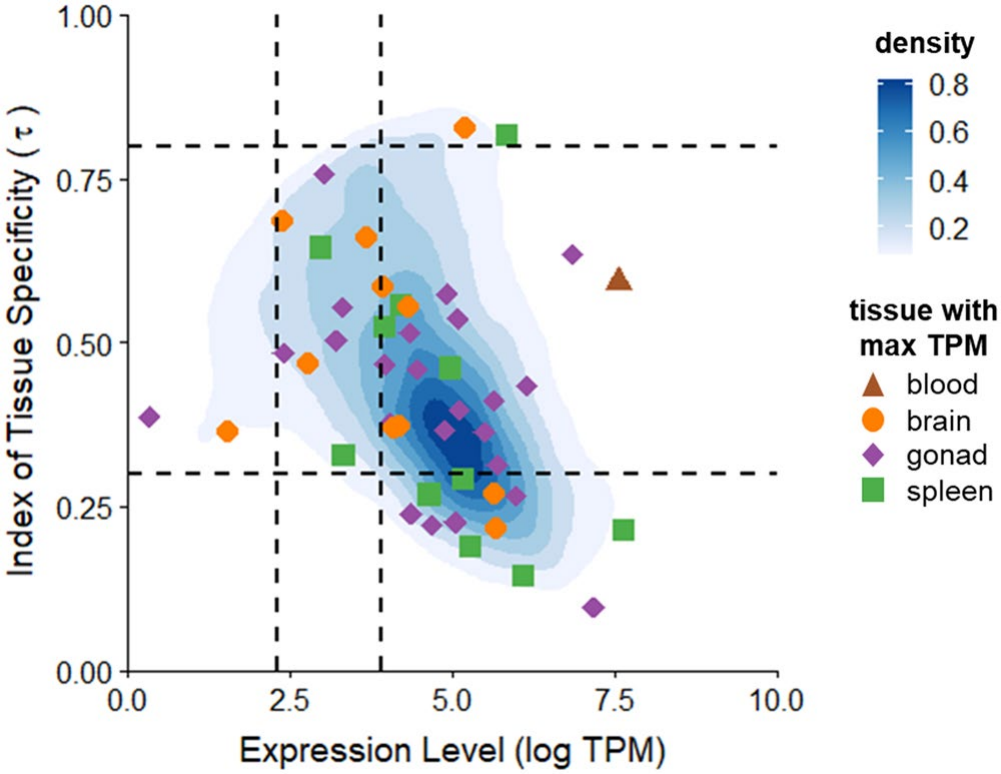
Correlation of summed expression levels (log TPM) across all tissues and index of tissue specificity (τ). Genes under positive selection are depicted as points (color and shape indicate the tissue in which each gene was maximally expressed). All other genes are shown as 2D kernel density estimates. Dashed lines create quadrats that denote expression values on the x axis (low, TPM < 10; medium, TPM = 10–50; high, TPM > 50) and τ on the y axis (broadly expressed, τ < 0.3; moderate, τ = 0.3–0.8; highly specific, τ > 0.8).

Expression levels (reported as transcripts-per-million, TPM) were highly variable among tissues and genes (median = 105.0 TPM; mean = 404.8 TPM ± 5348.9 SD). The summed expression level (log TPM) for a given gene across tissues was negatively correlated with the index of tissue specificity (τ) (Spearman ρ = −0.40, p < 0.0001; Fig. 3), suggesting that highly expressed genes are less tissue specific. However, tissue specificity also varied based on which tissue maximally expressed the gene (ANOVA, F_5,14617_ = 204.1, p < 0.0001). The lowest τ was found in genes that had their highest expression in blood, while the highest τ was found in genes with maximal expression in brain and liver (Table 2).

**Table 2:** Mean index of tissue specificity of expression (τ) for genes with maximal expression in each of the six tissues, together with 95% confidence intervals (CI). N_max_ represents the number of genes with maximal expression in each of the tissues.

Tissue	N_max_	τ_avg_	95% CI (τ)
Liver	847	0.522	0.510–0.534
Brain	3977	0.520	0.515–0.526
Muscle	413	0.459	0.438–0.480
Ovary	5507	0.445	0.441–0.449
Spleen	3127	0.417	0.412–0.423
Blood	752	0.388	0.377–0.398

### Positive selection analysis

To identify candidate genes under positive selection, we compared protein-coding sequences in the tree swallow with 8 other avian species. The branch-site test in PAML^46,47^ identified 46 genes with significant evidence of positive selection in the lineage leading to tree swallows using two different codon alignment methods (see Methods). Although there were no significantly overrepresented GO terms for this set, several genes were associated with processes that are relevant in the context of tree swallow life history or ecology, including feeding behavior (RMI1, CNRIP1), lipid metabolic processes (DECR2, LIPT1), reproduction (ALKBH5, PTTG1, EIF2B4, DRC7, CCDC40), immune system processes (IL12B, TRIM25, USP14, CPPED1, ICOS, TSPAN2), muscle system process (GTF2IRD1), and visual perception (FAM161A). The complete list of positively selected genes is in Supplementary Table S8.

We next explored whether accelerated molecular evolution of these proteins was associated with expression levels or tissue specificity. Similar to the overall patterns reported above, the summed expression level (log TPM) for positively selected genes was also negatively correlated with τ (ρ = −0.37, p = 0.01; Fig. 3). Positively selected genes tended to be expressed at lower levels across tissues (260.08 TPM, 95% CI = 126.33–393.83) compared with non-positively selected genes (405.23, 95% CI = 318.26–492.20; p = 0.08, by bootstrapping), but they did not differ in their tissue specificity (positively selected genes: τ = 0.434, 95% CI = 0.382–0.486 vs. non-positively selected genes: τ = 0.460, 95% CI = 0.457–0.463; p = 0.33). Two positively selected genes were highly tissue specific in their expression, cannabinoid receptor interacting protein 1 (CNRIP1) and inducible T-cell costimulator (ICOS), which had maximal expression in the brain and spleen, respectively (Fig. 3). Expression levels of positively selected genes did vary significantly across tissues (ANOVA, F_5_ = 3.27, p < 0.01), with ovary expressing significantly higher levels of these genes (average = 84.35 TPM, 95% CI = 44.82) than muscle (24.53 TPM, 95% CI = 20.87) or liver (27.50 TPM, 95% CI = 20.69), which expressed the lowest levels (Tukey test, p = 0.02).

### Tissue-specific capacity for steroid synthesis and sensitivity

**Figure 4:**
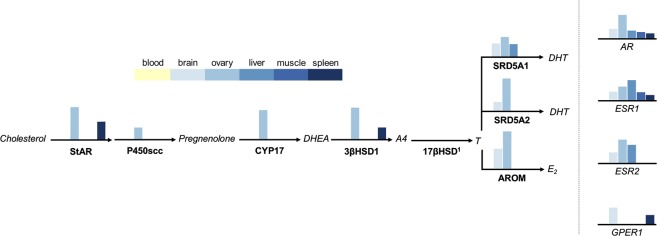
Steroidogenic capacity and sensitivity by tissue. The y-axis values are held constant throughout but are arbitrary as only relative expression levels are of importance. ^1^17βHSD data were excluded because of uncertainty around isoform function (see text). Abbreviations: StAR, steroidogenic acute regulatory protein; P450scc, cytochrome P450 side-chain cleavage; CYP17, cytochrome P450 17α-hydroxylase/17,20lysase; DHEA, dehydroepiandrosterone; 3βHSD1, 3β-hydroxysteroid dehydrogenase/isomerase; A4, androstenedione; 17βHSD, 17β-hydroxysteroid dehydrogenase; T, testosterone; SRD5A1, steroid 5 alpha-reductase 1; SRD5A2, steroid 5 alpha-reductase 2; AROM, cytochrome P450 aromatase; DHT, dihydrotestosterone; E_2_, 17β-estradiol; AR, androgen receptor; ESR1, estrogen receptor alpha; ESR2, estrogen receptor beta; GPER1, G protein-coupled estrogen receptor 1.

We further explored tissue-specific expression patterns of steroidogenic genes because of the emerging endocrine research on the role of different tissues in mediating sex steroid-related phenotypes. Our transcriptome included the major enzymes involved in sex steroid synthesis (StAR, p450scc, CYP17, 3βHSD1; see 17βHSD analysis below), although most were limited in their expression to the ovary and occasionally spleen (all τ > 0.63), with CYP17 being the most ovary specific (τ = 0.84) followed by 3βHSD1 (τ = 0.78). P450scc expression was generally low (max = 5.2 TPM), and MiSeq detected very low 3βHSD1 expression in brain and liver (2.0 and 1.7 TPM, respectively), suggesting only limited *de novo* steroidogenic potential outside of the ovary and spleen. Enzymes involved in later sex steroid conversion were primarily expressed in the brain and ovary, with SRD5A2 and AROM being predominantly ovary specific (τ = 0.81 and 0.79, respectively) and SRD5A1 less so (τ = 0.55). Sex steroid binding capabilities, on the other hand, were more ubiquitous; most sex steroid receptors examined (e.g. AR and ESR1) were expressed across most tissues (excluding blood), with GPER1 being more specific to the brain (all τ < 0.66). Thus, tissue specificity appeared to decrease along the steroidogenic pathway, from steroid synthesis to receptor binding (Fig. 4).

We excluded 17βHSD from the above analysis because it has several isoforms that both synthesize and metabolize sex steroids, and there is some suggestion that avian isoforms may function differently than the more well-characterized mammalian isoforms^48,49^. To ameliorate this uncertainty regarding pathways of sex steroid synthesis in birds, we identified 7 isoforms of 17βHSD and clustered them into two groups based on tissue-level expression patterns: cluster 1 was more ovary-specific and included 17βHSD1, 17βHSD2, and 17βHSD8; and cluster 2 had more general expression and included 17βHSD4, 17βHSD7, 17βHSD12, and 17βHSD13 (Supplementary Fig. S3). The most tissue-specific isoforms (17βHSD1, τ = 0.74, and 17βHSD2, τ = 0.79) were expressed most highly in the ovary, the primary site of sex steroidogenesis, while all other isoforms ranged from τ = 0.34–0.58.

## Discussion

Transcriptomic data from multiple tissues can shed light on important questions in evolutionary and organismal biology. Tree swallows are one of the most studied free-living birds across many disciplines within ecology, evolution, and behavior, and the *de novo* transcriptome assembly here provides a key molecular resource for further advancing these fields. Using this new resource, we first identified several dozen genes showing signs of positive selection along the tree swallow lineage, many of which are related to metabolic, reproductive, and immune processes. We also identified tissue-specific functions, while highlighting the potential for various tissues to locally produce and bind sex steroids. Below, we discuss the implications of these findings and how they inform our understanding of molecular and organismal evolution.

Validations suggest this transcriptome covers a wide range of protein-coding sequences, including 91.7% of BUSCOs thought to be found in all metazoans. This compares quite favorably to other *de novo* transcriptomes in passerines using BUSCO to evaluate completeness against the vertebrate gene set (30–62% complete)^50^. Additionally, our TransRate score of 0.38 is higher than > 50% of the transcriptomes deposited in the NCBI Transcriptome Shotgun Assembly database^44^. Finally, our backmapping rate of 82% is also comparable to other *de novo* transcriptomes in birds, including European starlings (*Sturnus vulgaris*, 82%)^50^ and rock doves (*Columba livia*, 70–80%)^35^. In addition, the 14,717 annotated transcripts closely match the 15,183 protein-coding genes identified in the *Parus major* assembly (Parus_major1.0.3), which shared the highest degree of sequence similarity to our data. This number is also similar to other *de novo* transcriptomes in passerines (ranges from 7,135–17,898; Table S2) and well-established reference transcriptomes in songbird species, like the zebra finch (*Taeniopygia guttata;* taeGut3.2.4) and collared flycatcher (*Ficedula albicollis;* FicAlb_1.4), which contain 17,488 and 15,303 annotated protein-coding genes, respectively. Subsequent studies would ideally include samples from males, non-breeding stages, and juveniles; however, data thus far suggest that this transcriptome from 2 females at 2 breeding stages (6 tissues each) has good coverage.

Tissue-specific gene expression provides one mechanism by which the same genome can generate differentiated phenotypes among tissues. In our dataset, uniquely expressed genes were largely considered typical for each tissue. For example, muscle-specific genes were associated with muscle contraction and spleen-specific genes with immune response. Two tissues, blood and liver, had few unique genes and no overrepresented GO terms, indicating they may perform more diverse functions. However, genes with maximum expression in the liver had relatively high tissue-specificity, and unique genes in the liver were related to processes like complement system, lipoprotein production (presumably for eggs), and metabolism (e.g., glucuronidation). The brain had the most uniquely expressed genes which were associated with synaptic transmission, neuropeptide signaling, and other neural processes. Furthermore, genes maximally expressed in the brain were relatively tissue-specific, suggesting the brain is enriched with differentiated cell types and/or unique functionality. This finding is supported by studies in mammals indicating the brain may have more fine-tuned expression networks than other tissues^51–53^. The ovary expressed the second highest number of unique genes and showed the most significant overrepresentation for terms pertaining to gene silencing, likely having to do with germ cell development. We also explored the shared gene expression patterns between ovary and brain, which had the greatest gene overlap. While shared expression profiles do not necessarily imply similar function^54^, these tissues shared genes associated with processes like hormone secretion and aggression, consistent with the well-established coordination of these tissues via the hypothalamic-pituitary-gonadal, or HPG axis, which mediates many reproductive and aggressive behaviors.

In an initial application of this transcriptome, we identified 46 putative positively selected genes in the lineage leading to tree swallows, compared to 8 other avian species. While we did not find any significantly overrepresented processes among these apparently rapidly evolving genes, GO terms associated with these genes represent potentially important and well-studied adaptations in this system. For example, tree swallows are the only aerial insectivore in our analysis (data for other swallows and swifts is currently being generated^55–57^). Aerial insectivores can spend 80% or more of the day in flight^5^, gathering prey during acrobatic flight, potentially requiring enhanced muscle functioning and visual perception, but they must also contend with periods of food shortages, which they do by adjusting growth to environmental conditions^58–61^. This metabolic flexibility could relate to positively selected genes involved in feeding behavior and lipid metabolism, such as RMI1, which regulates feeding behavior and energy homeostasis^62^, and CNRIP1, a highly brain-specific gene that modulates appetite through cannabinoid receptor 1 activity^63^. Genes related to fat metabolism generally appear to be more susceptible to rapid evolution along the passerine lineage^23^. We also found several genes associated with immune function that are potentially under positive selection, such as ICOS, a highly spleen-specific gene, associated with enhanced T-cell responses. T-cell functioning is important for tree swallows because their diet of emergent aquatic insects makes them more susceptible to the bioaccumulation of toxins that can reduce immune responsiveness^28^. Our selection analysis also identified genes associated with sperm performance (CCDC40 and DRC7)^64^ that could be related to the high rates of extra-pair paternity in tree swallows^31,33^, which is known to create strong selection pressures associated with sperm competition^65–67^. Therefore, many of the candidate genes under positive selection can be explained by well-studied adaptations specific to tree swallow life history and ecology.

Several factors interact to influence adaptive molecular evolution, including protein function, expression level, tissue specificity, and more^19^. Of these factors, the effects of expression level and tissue specificity are particularly well suited to transcriptomic analyses. For instance, more rapidly evolving genes tend to have lower expression, potentially due to selection against protein misfolding^19^. Our finding that genes under positive selection tended to be expressed at a lower level compared to the rest of the transcriptome supports the idea that fast-evolving genes are characterized by lower expression levels^20,22^. Furthermore, we found that expression levels were negatively correlated with tissue specificity, suggesting genes with more specific functions have lower expression levels^20,21,34^. Higher tissue specificity may act to release genes from evolutionary constraints by allowing for greater compartmentalization and avoiding potentially competing roles^19^. Specifically, genes expressed primarily in immune and reproductive tissues have been shown to be under strong selection^20,21,68,69^. While we did not find that positively selected genes were significantly more tissue specific, our analyses highlighted many positively selected genes related to reproduction (ALKBH5, PTTG1, EIF2B4, DRC7, CCDC40) and immune system processes (IL12B, TRIM25, USP14, CPPED1, ICOS, TSPAN2). Positively selected genes were also most abundant in the ovary, and two highly tissue-specific positively selected genes were expressed in the brain and spleen. Thus, genes in these tissues may be more rapidly evolving in tree swallows, which is consistent with their high rate of extra-pair paternity^31,33^ and propensity to accumulate environmental contaminants^27^. However, these conclusions should be regarded conservatively, because there are likely other genes under positive selection that we did not detect due to the species we included. For example, the cavity-nesting species (*Ficedula albicollis*, *Parus major*, *Cyanistes caeruleus*, and *Sturnus vulgaris*) shared a high degree of protein similarity with the tree swallow, likely preventing us from finding unique protein changes associated with adaptations required for cavity nesting (e.g., heightened territorial aggression). As more high-quality transcriptomes are developed for non-model organisms, we can begin to more accurately address these evolutionary questions.

In a second application of our transcriptome, we analyzed tissue-level variability along the pathway of sex steroid hormone production and binding as a window into the organismal potential for independent regulation of suites of sex steroid-mediated traits. The degree to which tissues can independently regulate this has become a hot topic in evolutionary endocrinology, due to the potential to decouple hormones from their organism-wide pleiotropic effects and elicit more specific, potentially adaptive suites of traits^37,70,71^. Recent work suggests that this kind of hormonal and behavioral plasticity may be particularly important for females, potentially allowing regulation of sex steroids, like testosterone, in a tissue-specific manner to avoid the systemic costs of elevated testosterone^15,41^. We found that steroid receptors were expressed almost ubiquitously across tissues (excluding blood), suggesting that most tissues studied here have the potential to locally regulate steroid sensitivity. This finding is not surprising given the dynamic functions steroids perform^72^, and past studies showing expression of these genes within the tissues included here^35,73–75^. Fewer studies, however, have measured expression of steroidogenic enzymes, particularly in peripheral tissues^15,74–76^. We found that these enzymes were primarily ovary-specific in gene expression, but as you move further along the steroidogenic pathway from *de novo* synthesis to later metabolism, genes became less tissue specific in their expression. The spleen was the only other tissue to express a sex steroid-synthesizing enzyme; however, it was 3βHSD1, which can be co-opted to synthesize corticosterone or other immunosteroids known to respond to metabolic and immune stressors^74,77^. Strikingly, steroid-synthesizing enzymes early in the pathway were largely absent from brain expression profiles. This is surprising considering that both male and female birds typically express enzymes capable of *de novo* steroid synthesis in the brain^48,78–80^. One possibility is that we failed to detect genes expressed at very low levels, and had we measured protein levels, which tend to be considerably higher than mRNA expression levels^79^, we would have found these genes in more tissues. Another possibility is that neurosteroid synthesis varies seasonally in its expression and our lack of non-breeding females precluded us from finding these genes in the brain. Enzymes capable of neurosteroid synthesis may have higher neural activity during the non-breeding season^80^, which could be indicative of a seasonal switch in how sex steroids are regulated. Future work with more biological replication is needed to fully tease apart these alternatives, though our findings clearly show marked sex steroid processing and binding abilities across many different tissues.

Components of the steroid pathway are still unresolved in birds, specifically for 17βHSD, which has multiple isoforms with unknown expression and/or function. Thus, as a final application of this transcriptome, we used patterns of tissue-specific gene expression to improve knowledge of putative 17βHSD functionality. We were unable to identify all known mammalian isoforms, an issue also addressed in the zebra finch genome assembly^48^. Of the 14 known 17βHSD isoforms^81^, we found 7 in our transcriptome. The two isoforms responsible for testosterone synthesis in most mammals (17βHSD3 and 17βHSD5)^49^ were not present in the current transcriptome. Within the zebra finch genome assembly, 17βHSD5 was also not found and 17βHSD3 was localized to the Z chromosome^48^. These findings could stem from low sequence homology, or birds may not have or express all known steroidogenic enzymes or they may synthesize testosterone with different isoforms than mammals. In general, the role of 17βHSD enzymes are controversial because they can be species-specific and act on a large set of substrates (e.g., steroids, fatty acids, retinols, etc.), so their function is partly defined by tissue expression^81^. We found three isoforms that were primarily expressed in the ovary and had putative roles with steroid processes, including estradiol synthesis (17βHSD1) and steroid inactivation (17βHSD2 and 17βHSD8) in mammals^49^. Of these, 17βHSD1 is the most likely candidate to perform testosterone synthesis in female tree swallows. It was largely ovary-specific (τ = 0.74) and has demonstrated the ability to synthesize both estrogen and testosterone in other species (e.g., mice^82^). Regardless, this highlights the need for future studies on 17βHSD in birds, particularly 17βHSD1, which our data implicate as a key steroidogenic isoform.

## Methods

### Sample collection

Tissues were collected from adult female tree swallows during territory establishment early in the breeding season (n = 1 female) and during incubation (n = 1). Collections occurred in Monroe and Brown County, Indiana (39°9 N, 86°31 W) in April and May 2016 between 900–1200. Females were euthanized with an overdose of isoflurane, followed by decapitation, and tissues were immediately collected. Gonad, liver, spleen, pectoral muscle, trunk blood, and brain were frozen on powdered dry ice and transferred to −80 °C in the lab. The female collected during territory establishment was actively engaging in aggressive interactions at an empty nest box (KAR, pers. obs.) and had recrudesced ovaries with small white follicles. The female collected in May was incubating a clutch of 5 eggs completed 3 days earlier; she had mostly white ovarian follicles with approx. 4 small yellow follicles. While the use of only two adult females does limit our ability to detect some genes (e.g., genes only expressed in juveniles or males), it helps reduce concerns about integrating allelic variation thereby improving the assembly. This study was approved by the Bloomington Institutional Animal Care and Use Committee under protocol #15–004 and all methods were performed in accordance with the relevant guidelines and regulations.

### RNA extraction, library preparation, and sequencing

Total RNA was extracted from each sample separately using the phenol-chloroform-based Trizol method, following the manufacturer’s instructions (Invitrogen, Carlsbad, CA). Total RNA was resuspended in water, and quality (RIN > 8.0) and quantity of RNA was analyzed with an Agilent 2200 TapeStation (Agilent Technologies, Santa Clara, CA). Total RNA was prepared into equimolar pools for each tissue and submitted to Indiana University’s Center for Genomics and Bioinformatics for cDNA library construction using a TruSeq Stranded mRNA LT Sample Prep Kit (Illumina) following the standard manufacturing protocol. When preparing larger fragments, the fragmentation step was reduced from 8 min to 10 s at 94 °C. Sequencing was performed by using an Illumina NextSeq 500/550 Kit v2 with a 150-cycle sequencing module generating 81 bp paired-end reads. After the sequencing run, demultiplexing was performed with bcl2fastq v2.20.0.422. We additionally performed sequencing using a MiSeq Kit v3 with a 600-cycle sequencing module generating 305 bp paired-end reads. These longer reads were used to improve confidence in our assembly.

### Assembly and annotation

Trimmomatic (version 0.36)^83^ was used to trim reads and to remove adapter sequences and low-quality reads. The transcriptomes were assembled using Trinity (version 2.6.5) and spades (version 3.11.1) with a minimum contig length of 100. The spades assemblies were performed with a k of 35, 55, and 71. The different assemblies were then compared and merged using the EvidentialGene pipeline (http://arthropods.eugenes.org/EvidentialGene/trassembly.html). The final merged assembly consisted of 207,739 transcripts. These transcripts were then searched against several reference genomes, including *Homo sapiens*, *Gallus gallus*, *Parus major*, *Serinus canaria*, *Sturnus vulgaris*, *Ficedula albicollis*, *Zonotrichia albicollis*, *Taeniopygia guttata*, *Geospiza fortis*, and *Chaetura pelagica* to identify potential homologous proteins using NCBI BLAST (version 2.2.26), resulting in 144,119 transcripts with significant BLAST hits (e < 1e-10). The peptide alignment information was used to identify discrete protein-coding segments with at least 70% identity that also covered at least 50% of the best matching full-length protein (n = 22,825 transcripts). Putatively unspliced introns were removed from the putative protein-coding segments. Finally, largely redundant transcripts were removed using cd-hit-est (version 4.6.8)^84,85^. This resulted in a set of high confidence protein-coding segments (n = 14,717) that could be used both for phylogenetic comparisons as well as abundance measurements. Reads from each tissue (NextSeq and MiSeq) were mapped against the protein-coding portions of the transcriptome using bwa mem (version 0.7.17). We assessed assembly quality and accuracy using TransRate^44^ (including backmapping rate), and we assessed assembly completeness using BUSCO^45^ (lineage dataset = aves_odb9; 40 species; 4915 conserved genes).

Transcripts were converted to their associated gene name using bioDBnet (https://biodbnet-abcc.ncifcrf.gov/db/db2db.php) and functional annotations were retrieved from the GO database (version 1.2). Transcripts with TPM values of less than 1 were considered absent from a tissue (n = 14,623 transcripts had a TPM ≥ 1 in at least one tissue) and we further characterized gene expression levels as low expression = TPM < 10, medium expression = 10 < TPM < 50, and high expression = TPM > 50^86^.

### Index of tissue specificity

We calculated an index of tissue specificity of gene expression (τ)^54^, using the methods presented in Mank *et al*.^87^. In order to reduce the effect of sampling stochasticity from genes with low expression, TPM was set to 2 to account for tissues with no detected expression. The range of τ for a gene is between 0 and 1; highly tissue-specific transcripts have values close to one (τ > 0.8) and widely expressed transcripts (e.g., housekeeping genes) have lower values (τ < 0.3)^54^. We explored the accuracy of these τ cut-offs by comparing the distribution of the most abundant and unique gene in each tissue (Supplementary Table S3) with well-known housekeeping genes, including SDHA, UBC, GAPDH, RPL4, HMBS, and ACTB, many of which are commonly used in birds^88,89^. The distributions were significantly different (Kolmogorov-Smirnov test: p = 0.002) as the unique genes ranged from τ = 0.80–0.89 and the housekeeping genes ranged from τ = 0.15–0.30, supporting the proposed cut-offs.

### Characterizing tissue expression profiles

To characterize patterns of gene expression unique to each tissue, we compared transcript presence/absence across all tissues using the UpSetR package^90^ and the 10 most abundant, unique genes were identified in each tissue. To clarify processes unique to each tissue, transcripts expressed in only a single tissue were subjected to a GO overrepresentation analysis (see below). We additionally identified steroidogenic enzymes (StAR, P450scc, CYP17, 3βHSD1, SRD5A1, and AROM) and receptors (androgen and estrogen receptors) expressed in each tissue to evaluate tissue-specific steroidogenic capacity and sensitivity. We also analyzed the multiple 17βHSD isoforms by clustering them based on tissue expression using Euclidean distance.

### Positive selection analysis

To compare patterns of protein coding sequence evolution along the lineage leading to the tree swallow we downloaded the peptides and coding nucleotide sequences on July 27, 2018 for the 7 other avian species belonging to the order Passeriformes on the NCBI database (white-throated sparrow, *Zonotrichia albicollis*; American crow, *Corvus brachyrhynchos*; great tit, *Parus major*; collared flycatcher, *Ficedula albicolllis*; zebra finch, *Taeniopygia guttata*; blue tit, *Cyanistes caeruleus*; and European starling, *Sturnus vulgaris*), along with the chicken (*Gallus gallus*) to serve as an outgroup (Supplementary Table S9). Sequences were filtered such that only the longest isoform of each gene was retained. The filtered protein sequences from these 8 species plus the predicted peptides for the tree swallow generated here were clustered into orthologous groups using an all-v-all BLAST search^91^ that generated e-values used to inform the main clustering criterion for the MCL program^92^. Of the resulting groups of orthologous transcripts, we identified 3,015 single-copy peptide groups that have exactly one copy of the gene present in each species. We aligned these with two codon alignment programs (PRANK^93^ and MACSE^94^) allowing us to look for overlapping genes in the final list of genes under positive selection as a means to eliminate possible errors due to poor alignments. Alignments were also masked with GBlocks^95^ to remove poorly aligned or gap-ridden positions. We reconstructed gene trees with RAxML^96^ which were used to infer a species tree with ASTRAL (Supplementary Fig. S4)^97^. To identify genes evolving under positive selection along the tree swallow lineage (orange branch in Supplementary Fig. S4), we set that branch of the phylogeny as the foreground branch in a PAML^46^ branch-site test^47^. However, because we observed high amounts of discordance between individual gene trees and the inferred species tree (incomplete lineage sorting; ILS) in our phylogeny, we ran the branch-site test on the coding sequence of each gene using the gene tree inferred from the coding sequence of that locus, rather than the inferred species tree for all loci. This helps minimize the effect of substitutions produced by ILS^98^. For detailed methods and a discussion of the phylogenetic methods see supplementary information (Supplementary Methods: Positive Selection Analysis). To better characterize genes under positive selection, we performed a GO overrepresentation analysis (see below) and explored relationships between positive selection, gene expression levels, and tissue specificity.

### GO overrepresentation analyses

To investigate the functions of genes uniquely expressed in each tissue and genes under positive selection, we performed GO overrepresentation analyses^99,100^. We assessed the complete biological processes, molecular functions, and cellular components using *Gallus gallus* as the background reference and a Fisher’s Exact test with FDR correction. Only GO terms with ≥3 genes are considered.

## Supplementary information

Supplementary Materials

## Data Availability

Raw sequence reads can be obtained from the Gene Expression Omnibus database (GEO accession number GSE126210). The transcriptome has been submitted to the Transcriptome Shotgun Assembly project and deposited at DDBJ/EMBL/GenBank under the accession GHGE00000000. The version described in this paper is the first version, GHGE01000000.
