# Supplementary material for: Tissue-specific expression profiles and positive selection analysis in the tree swallow (Tachycineta bicolor) using a de novo transcriptome assembly
Source: Sci Rep. 2019 Nov 1;9:15849. doi: 10.1038/s41598-019-52312-4 (PMC6825141; doi:10.1038/s41598-019-52312-4)
Supplement: Supplementary file 1 — Supplementary Materials [file 41598_2019_52312_MOESM1_ESM.doc]

**Supplementary information**: **Tissue-specific expression profiles and positive selection analysis in the tree swallow (*Tachycineta bicolor*) using a *de novo* transcriptome assembly**

Alexandra B. Bentz^1,2*^, Gregg W.C. Thomas^1,3^, Douglas B. Rusch^1,4^, and Kimberly A. Rosvall^1,2^

^1^Department of Biology, Indiana University, Bloomington, IN 47405, USA

^2^Center for the Integrative Study of Animal Behavior, Indiana University, Bloomington, IN 47405, USA

^3^Department of Computer Science, Indiana University, Bloomington, IN 47405, USA

^4^Center for Genomics and Bioinformatics, Indiana University, Bloomington, IN 47405 USA

Corresponding author^*^: bentza@iu.edu (ABB)

**Supplementary Table S1**. Number of paired-end reads detected in each tissue sample using RNA sequencing.

| **Sample** | **Total Reads**  **(NextSeq)** | **Total Reads**  **(MiSeq)** |
| --- | --- | --- |
| Brain | 118,720,515 | 2,164,333 |
| Blood | 167,150,038 | 2,991,969 |
| Gonad | 121,449,498 | 2,311,257 |
| Liver | 140,001,405 | 2,544,860 |
| Muscle | 139,585,689 | 2,419,924 |
| Spleen | 113,626,107 | 2,218,625 |

| **Species** | **Total assembled transcripts** | **GC (%)** | **N50 length (bp)** | **Median length (bp)** | **Mean length (bp)** | **Filtered annotated transcripts** | **No. of tissues** |
| --- | --- | --- | --- | --- | --- | --- | --- |
| *Tachycineta bicolor* (tree swallow) | 207,739 | 45.87 | 1,889 | 809 | 1,343 | 14,717 | 6 |
| *Sturnus vulgaris* (European starling)^1^ | 59,557 | 48.28 | 1,765 | 626 | 1,091 | 17,898 | 1 |
| *Melospiza melodia* (song sparrow)^2^ | 276,670 | - | 4,072 | - | 1,416 | 16,864 | 1 |
| *Zonotrichia gambelii* (white-crowned sparrow)^2^ | 307,617 | - | 3,415 | - | 1,591 | 16,646 | 1 |
| Setophaga caerulescens (black‐throated blue warbler)^3^ | - | - | 2,107 | - | 977 | 16,261 | 4 |
| Paradoxornis bulomachus (vinous-throated parrotbill)^4^ | 465,613 | - | - | - | - | 16,028 | 2 |
| *Zonotrichia albicollis* (white-throated sparrow)^2^ | 149,184 | - | 2,284 | - | 982 | 15,805 | 1 |
| *Turdus merula* (common blackbird)^5^ | 162,158 | - | - | - | 964 | 15,580 | 14 |
| *Haemorhous mexicanus* (house finch)^6^ | 222,678 | - | - | 347 | 827 | 9,646 | 1 |
| *Chloris chloris* (European greenfinch)^7^ | 66,084 | - | 803 | - | 596 | 7,135 | 1 |
| *Andropadus virens* (little greenbul)^8^ | 286,494 | 45.48 | 1,619 | - | 815 | - | 3 |

**Supplementary Table S2.** Assembly statistics for *de novo* assembled passerine transcriptomes using Illumina sequencing technology. The tree swallow assembly presented in the current study (highlighted in grey) compares favorably with recent studies. Studies are ordered by number of annotated transcripts. A dash (-) denotes that the information was not reported. See footnotes for references.

1. Richardson, M. F., Sherwin, W. B. & Rollins, L. A. *De novo* assembly of the liver transcriptome of the European starling, *Sturnus vulgaris*. *J. Genomics* **5**, 54-57 (2017).

2. Balakrishnan, C. N. *et al.* Brain transcriptome sequencing and assembly of three songbird model systems for the study of social behavior. *PeerJ* **2**, e396 (2014).

3. Kaiser, S. A. *et al.* A comparative assessment of SNP and microsatellite markers for assigning parentage in a socially monogamous bird. *Mol. Ecol. Res.* **17**, 183–193 (2017).

4. Chu, J.-H., Lin, R.-C., Yeh, C.-F., Hsu, Y.-C. & Li, S.-H. Characterization of the transcriptome of an ecologically important avian species, the vinous-throated parrotbill *Paradoxornis webbianus bulomachus* (Paradoxornithidae; Aves). *BMC Genomics* **13**, 149 (2012).

5. Koglin, S., Trense, D., Wink, M., Sauer-Gürth, H. & Tietze, D. T. Characterization of a de novo assembled transcriptome of the common blackbird (*Turdus merula*). *PeerJ* **5**, e4045 (2017).

6. Zhang, Q., Hill, G. E., Edwards, S. V. & Backström, N. A house finch (*Haemorhous mexicanus*) spleen transcriptome reveals intra-and interspecific patterns of gene expression, alternative splicing and genetic diversity in passerines. *BMC Genomics* **15**, 305 (2014).

7. Meitern, R., Andreson, R. & Hõrak, P. Profile of whole blood gene expression following immune stimulation in a wild passerine. *BMC Genomics* **15**, 533 (2014).

8. Zhen, Y. *et al.* Genomic divergence across ecological gradients in the Central African rainforest songbird (*Andropadus virens*). *Mol. Ecol.* **26**, 4966–4977 (2017).

**Supplementary Table S3**. Top 10 unique genes per tissue (TPM < 1 in all other tissues). Uncharacterized proteins are excluded and ‘Symbol’ denotes human orthologs.

| Tissue | Symbol | Name | TPM |
| --- | --- | --- | --- |
| Ovary | ZP2 | zona pellucida sperm-binding protein 2 | 796.1 |
|  | HTA2 | histone H2A-beta, sperm-like | 566.4 |
|  | BIRC7 | baculoviral IAP repeat-containing protein 7 isoform X1 | 527.4 |
|  | PLIN3 | perilipin-3-like isoform X1 | 413.6 |
|  | MOS | proto-oncogene serine/threonine-protein kinase mos | 398.4 |
|  | THAP8 | THAP domain-containing protein 8-like | 304.5 |
|  | NEIL2 | endonuclease 8-like 2 | 297.0 |
|  | ZP4 | zona pellucida sperm-binding protein 4 | 295.6 |
|  | NANOG | homeobox protein NANOG | 277.7 |
|  | H1FOO | histone H1oo | 242.0 |
| Brain | PLP1 | myelin proteolipid protein isoform X1 | 837.4 |
|  | SLC17A6 | vesicular glutamate transporter 2 | 636.8 |
|  | PLP1 | myelin proteolipid protein isoform X2 | 569.7 |
|  | SLC6A1 | sodium- and chloride-dependent GABA transporter 1 | 445.6 |
|  | GABRA1 | gamma-aminobutyric acid receptor subunit alpha-1 | 416.0 |
|  | SLC1A3 | excitatory amino acid transporter 1 | 271.6 |
|  | KIAA1211 | uncharacterized protein KIAA1211 homolog | 256.6 |
|  | FAIM2 | protein lifeguard 2 | 243.4 |
|  | MYT1L | myelin transcription factor 1-like protein isoform X5 | 220.4 |
|  | SLC32A1 | vesicular inhibitory amino acid transporter | 217.4 |
| Spleen | CXCL2 | C-X-C motif chemokine 2-like | 555.6 |
|  | MARCO | macrophage receptor MARCO | 376.2 |
|  | CXCR5 | C-X-C chemokine receptor type 5 | 150.7 |
|  | EVI2A | protein EVI2A | 124.7 |
|  | PAX5 | paired box protein Pax-5 isoform X1 | 115.9 |
|  | PIGR | polymeric immunoglobulin receptor-like | 97.0 |
|  | CD96 | T-cell surface protein tactile | 96.2 |
|  | FORF | formin-F-like | 93.1 |
|  | TARP | TCR gamma alternate reading frame protein | 86.2 |
|  | CD28 | T-cell-specific surface glycoprotein CD28 isoform X1 | 71.6 |
| Liver | C6 | complement component C6 isoform X1 | 138.9 |
|  | MSMB | beta-microseminoprotein-like | 124.4 |
|  | NR1H4 | bile acid receptor isoform X1 | 80.7 |
|  | AADAC | arylacetamide deacetylase | 80.2 |
|  | SLC2A2 | solute carrier family 2, facilitated glucose transporter… | 64.4 |
|  | DGAT2 | diacylglycerol O-acyltransferase 2-like | 56.2 |
|  | SLC51B | organic solute transporter subunit beta | 53.5 |
|  | APOV1 | apovitellenin-1-like isoform X1 | 53.4 |
|  | UGT1A1 | UDP-glucuronosyltransferase 1-1-like isoform X4 | 53.4 |
|  | UGT1A1 | UDP-glucuronosyltransferase 1-1-like isoform X1 | 42.7 |
| Muscle | TNNC2 | troponin C, skeletal muscle | 656.7 |
|  | CKMT2 | creatine kinase S-type, mitochondrial | 334.8 |
|  | MYLK4 | myosin light chain kinase family member 4 | 130.8 |
|  | CMYA5 | cardiomyopathy-associated protein 5 | 107.8 |
|  | APOBEC2 | C-_U-editing enzyme APOBEC-2 | 106.6 |
|  | FSD2 | fibronectin type III and SPRY domain-containing protein 2 | 77.4 |
|  | PPP1R3A | protein phosphatase 1 regulatory subunit 3A | 71.3 |
|  | SMPX | small muscular protein | 71.1 |
|  | MYOZ2 | myozenin-2 | 51.4 |
|  | AMPD1 | AMP deaminase 1 | 46.2 |
| Blood | TSPO2 | translocator protein 2 isoform X1 | 90.1 |
|  | HBP | hemoglobin subunit pi | 36.4 |
|  | NPFFR2 | neuropeptide FF receptor 2 isoform X2 | 10.7 |
|  | CRYGS | beta-crystallin S | 10.1 |
|  | ERVK-25 | endogenous retrovirus group K member 25 Pol protein-like | 7.1 |
|  | SLFN12 | schlafen family member 12-like | 6.3 |
|  | SPTBN5 | spectrin beta chain, non-erythrocytic 5 isoform X3 | 5.3 |
|  | AOC3 | membrane primary amine oxidase-like isoform X1 | 3.3 |
|  | NXT2 | NTF2-related export protein 2 isoform X3 | 2.5 |
|  | CYB5 | cytochrome b5 isoform X2 | 2.5 |

**Supplementary Table S4**. Results of a gene ontology (GO) statistical overrepresentation test for genes that are unique to the spleen (n=44 of 114 genes mapped to GO terms). The most specific terms with significant effects (Fisher’s adjusted p < 0.05) and that contain ≥3 genes are reported. Ont=Ontology (BP=Biological Process; MF = Molecular Function; CC = Cellular Component).

| **GO ID** | **Ont.** | **Total** | **Exp.** | **Obs.** | **Fold** | **FDR** |
| --- | --- | --- | --- | --- | --- | --- |
| immune response (GO:0006955) | BP | 485 | 1.17 | 10 | 8.57 | <0.001 |
| negative T cell selection (GO:0043383) | BP | 10 | 0.02 | 3 | >100 | 0.003 |
| regulation of lymphocyte proliferation (GO:0050670) | BP | 106 | 0.25 | 5 | 19.61 | 0.005 |
| positive regulation of interleukin-12 production (GO:0032735) | BP | 14 | 0.03 | 3 | 89.08 | 0.005 |
| leukocyte chemotaxis (GO:0030595) | BP | 69 | 0.17 | 4 | 24.1 | 0.013 |
| chemokine-mediated signaling pathway (GO:0070098) | BP | 46 | 0.39 | 5 | 12.83 | 0.02 |
| positive regulation of T cell activation (GO:0050870) | BP | 88 | 0.21 | 4 | 18.9 | 0.025 |
| regulation of immunoglobulin production (GO:0002637) | BP | 30 | 0.07 | 3 | 41.57 | 0.025 |
| positive regulation of immune effector process (GO:0002699) | BP | 95 | 0.23 | 4 | 17.5 | 0.03 |
| negative regulation of apoptotic process (GO:0043066) | BP | 466 | 1.12 | 7 | 6.24 | 0.037 |
| External side of plasma membrane (GO:0009897) | CC | 271 | 0.65 | 6 | 9.20 | 0.044 |
| C-C chemokine receptor activity (GO:0016493) | MF | 17 | 0.04 | 3 | 72.77 | 0.028 |
| C-C chemokine binding (GO:0019957) | MF | 16 | 0.04 | 3 | 77.32 | 0.048 |

**Supplementary Table S5**. Results of a gene ontology (GO) statistical overrepresentation test for genes that are unique to the muscle (n=27 of 46 genes mapped to GO terms). The most specific terms with significant effects (Fisher’s adjusted p < 0.05) and that contain ≥3 genes are reported. Ont=Ontology (BP=Biological Process; MF = Molecular Function; CC = Cellular Component).

| **GO ID** | **Ont.** | **Total** | **Exp.** | **Obs.** | **Fold** | **FDR** |
| --- | --- | --- | --- | --- | --- | --- |
| musculoskeletal movement (GO:0050881) | BP | 24 | 0.04 | 3 | 72.43 | 0.025 |
| smooth muscle contraction (GO:0006939) | BP | 34 | 0.06 | 3 | 51.13 | 0.039 |
| skeletal muscle tissue development (GO:0007519) | BP | 102 | 0.18 | 4 | 22.71 | 0.043 |
| M band (GO:0031430) | CC | 19 | 0.03 | 3 | 91.49 | 0.003 |
| actin binding (GO:0003779) | MF | 357 | 0.62 | 7 | 11.36 | 0.009 |

**Supplementary Table S6**. Results of a gene ontology (GO) statistical overrepresentation test for genes that are unique to the ovary (n=138 of 317 genes mapped to GO terms). The most specific terms with significant effects (Fisher’s adjusted p < 0.05) and that contain ≥3 genes are reported. Ont=Ontology (BP=Biological Process; MF = Molecular Function; CC = Cellular Component).

| **GO ID** | **Ont.** | **Total** | **Exp.** | **Obs.** | **Fold** | **FDR** |
| --- | --- | --- | --- | --- | --- | --- |
| gene silencing by RNA (GO:0031047) | BP | 53 | 0.43 | 6 | 14.06 | 0.005 |
| hindlimb morphogenesis (GO:0035137) | BP | 35 | 0.28 | 5 | 17.74 | 0.009 |
| regulation of transcription, DNA-templated (GO:0006355) | BP | 2260 | 18.2 | 38 | 2.09 | 0.009 |
| tissue development (GO:0009888) | BP | 1006 | 8.1 | 22 | 2.72 | 0.011 |
| cellular phosphate ion homeostasis (GO:0030643) | BP | 5 | 0.04 | 3 | 74.5 | 0.012 |
| gonad development (GO:0008406) | BP | 107 | 0.86 | 7 | 8.12 | 0.014 |
| oogenesis (GO:0048477) | BP | 44 | 0.35 | 5 | 14.11 | 0.015 |
| positive regulation of gene expression (GO:0010628) | BP | 1349 | 10.86 | 26 | 2.39 | 0.015 |
| embryonic organ morphogenesis (GO:0048562) | BP | 224 | 1.8 | 9 | 4.99 | 0.031 |
| forebrain morphogenesis (GO:0048853) | BP | 9 | 0.07 | 3 | 41.39 | 0.033 |
| male meiosis I (GO:0007141) | BP | 10 | 0.08 | 3 | 37.25 | 0.037 |
| single fertilization (GO:0007338) | BP | 58 | 0.47 | 5 | 10.7 | 0.040 |
| sensory organ morphogenesis (GO:0090596) | BP | 185 | 1.49 | 8 | 5.37 | 0.043 |
| Sequence-specific DNA binding (GO:0043565) | MF | 996 | 8.02 | 28 | 8.02 | <0.001 |
| DNA-binding transcription activator activity (GO:0001228) | MF | 239 | 1.92 | 11 | 5.71 | 0.004 |
| polysome binding (GO:1905538) | MF | 5 | 0.04 | 3 | 74.50 | 0.013 |

**Supplementary Table S7**. Results of a gene ontology (GO) statistical overrepresentation test for genes that are unique to the brain (n=219 of 442 genes mapped to GO terms). The most specific terms with significant effects (Fisher’s adjusted p < 0.05) and that contain ≥3 genes are reported. Ont=Ontology (BP=Biological Process; MF = Molecular Function; CC = Cellular Component).

| **GO ID** | **Ont.** | **Total** | **Exp.** | **Obs.** | **Fold** | **FDR** |
| --- | --- | --- | --- | --- | --- | --- |
| developmental induction (GO:0031128) | BP | 24 | 0.3 | 6 | 20.17 | <0.001 |
| transmission of nerve impulse (GO:0019226) | BP | 35 | 0.43 | 7 | 16.14 | <0.001 |
| positive regulation of synapse assembly (GO:0051965) | BP | 43 | 0.53 | 7 | 13.13 | <0.001 |
| potassium ion transmembrane transport (GO:0071805) | BP | 145 | 1.8 | 18 | 10.02 | <0.001 |
| locomotory behavior (GO:0007626) | BP | 137 | 1.7 | 15 | 8.83 | <0.001 |
| neuropeptide signaling pathway (GO:0007218) | BP | 86 | 1.07 | 9 | 8.44 | <0.001 |
| adenylate cyclase-activating G protein-coupled…(GO:0007189) | BP | 94 | 1.17 | 9 | 7.73 | <0.001 |
| axon guidance (GO:0007411) | BP | 158 | 1.96 | 13 | 6.64 | <0.001 |
| synapse organization (GO:0050808) | BP | 150 | 1.86 | 11 | 5.92 | <0.001 |
| regulation of synaptic transmission, glutamater…(GO:0051966) | BP | 34 | 0.42 | 6 | 14.24 | 0.001 |
| cell-cell adhesion via plasma-membrane adhesi…(GO:0098742) | BP | 167 | 2.07 | 11 | 5.31 | 0.001 |
| action potential (GO:0001508) | BP | 57 | 0.71 | 7 | 9.91 | 0.001 |
| protein homooligomerization (GO:0051260) | BP | 203 | 2.52 | 12 | 4.77 | 0.002 |
| oligodendrocyte differentiation (GO:0048709) | BP | 39 | 0.48 | 6 | 12.41 | 0.002 |
| regulation of neural retina development (GO:0061074) | BP | 10 | 0.12 | 4 | 32.27 | 0.002 |
| G protein-coupled serotonin receptor signaling…(GO:0098664) | BP | 25 | 0.31 | 5 | 16.14 | 0.003 |
| regulation of cytosolic calcium ion concentration (GO:0051480) | BP | 187 | 2.32 | 11 | 4.75 | 0.003 |
| regulation of neurological system process (GO:0031644) | BP | 45 | 0.56 | 6 | 10.76 | 0.003 |
| regulation of presynaptic membrane potential (GO:0099505) | BP | 3 | 0.04 | 3 | 80.68 | 0.003 |
| embryonic forelimb morphogenesis (GO:0035115) | BP | 27 | 0.33 | 5 | 14.94 | 0.003 |
| retina morphogenesis in camera-type eye (GO:0060042) | BP | 46 | 0.57 | 6 | 10.52 | 0.003 |
| regulation of neurotransmitter transport (GO:0051588) | BP | 78 | 0.97 | 7 | 7.24 | 0.006 |
| regulation of excretion (GO:0044062) | BP | 5 | 0.06 | 3 | 48.41 | 0.007 |
| negative regulation of amine transport (GO:0051953) | BP | 5 | 0.06 | 3 | 48.41 | 0.007 |
| cell proliferation in forebrain (GO:0021846) | BP | 17 | 0.21 | 4 | 18.98 | 0.008 |
| forebrain dorsal/ventral pattern formation (GO:0021798) | BP | 6 | 0.07 | 3 | 40.34 | 0.010 |
| regulation of neuroblast proliferation (GO:1902692) | BP | 20 | 0.25 | 4 | 16.14 | 0.013 |
| regulation of AMPA receptor activity (GO:2000311) | BP | 20 | 0.25 | 4 | 16.14 | 0.013 |
| dorsal/ventral axis specification (GO:0009950) | BP | 7 | 0.09 | 3 | 34.58 | 0.013 |
| postsynaptic neurotransmitter receptor diffusio…(GO:0098970) | BP | 7 | 0.09 | 3 | 34.58 | 0.013 |
| synaptic transmission, glycinergic (GO:0060012) | BP | 7 | 0.09 | 3 | 34.58 | 0.013 |
| muscle contraction (GO:0006936) | BP | 123 | 1.52 | 8 | 5.25 | 0.013 |
| regulation of neurotransmitter levels (GO:0001505) | BP | 198 | 2.45 | 10 | 4.07 | 0.015 |
| adenylate cyclase-inhibiting G protein-coupled…(GO:0007197) | BP | 8 | 0.1 | 3 | 30.26 | 0.017 |
| positive regulation of peptide hormone secretion (GO:0090277) | BP | 44 | 0.55 | 5 | 9.17 | 0.019 |
| negative regulation of neuron differentiation (GO:0045665) | BP | 131 | 1.62 | 8 | 4.93 | 0.019 |
| chondrocyte differentiation (GO:0002062) | BP | 70 | 0.87 | 6 | 6.92 | 0.020 |
| regulation of Wnt signaling pathway (GO:0030111) | BP | 206 | 2.55 | 10 | 3.92 | 0.020 |
| negative regulation of synaptic transmission (GO:0050805) | BP | 26 | 0.32 | 4 | 12.41 | 0.026 |
| glial cell development (GO:0021782) | BP | 48 | 0.59 | 5 | 8.4 | 0.026 |
| embryonic camera-type eye formation (GO:0060900) | BP | 10 | 0.12 | 3 | 24.21 | 0.026 |
| lens fiber cell differentiation (GO:0070306) | BP | 27 | 0.33 | 4 | 11.95 | 0.029 |
| telencephalon regionalization (GO:0021978) | BP | 11 | 0.14 | 3 | 22 | 0.033 |
| embryonic hindlimb morphogenesis (GO:0035116) | BP | 28 | 0.35 | 4 | 11.53 | 0.033 |
| regulation of long-term neuronal synaptic plasti…(GO:0048169) | BP | 11 | 0.14 | 3 | 22 | 0.033 |
| regulation of metal ion transport (GO:0010959) | BP | 183 | 2.27 | 9 | 3.97 | 0.034 |
| pituitary gland development (GO:0021983) | BP | 29 | 0.36 | 4 | 11.13 | 0.036 |
| memory (GO:0007613) | BP | 53 | 0.66 | 5 | 7.61 | 0.038 |
| cell fate specification involved in pattern specif…(GO:0060573) | BP | 12 | 0.15 | 3 | 20.17 | 0.039 |
| regulation of short-term neuronal synaptic plast…(GO:0048172) | BP | 12 | 0.15 | 3 | 20.17 | 0.039 |
| excitatory postsynaptic potential (GO:0060079) | BP | 54 | 0.67 | 5 | 7.47 | 0.040 |
| positive regulation of ion transport (GO:0043270) | BP | 118 | 1.46 | 7 | 4.79 | 0.043 |
| cell fate determination (GO:0001709) | BP | 31 | 0.38 | 4 | 10.41 | 0.043 |
| eye photoreceptor cell differentiation (GO:0001754) | BP | 31 | 0.38 | 4 | 10.41 | 0.043 |
| positive regulation of amine transport (GO:0051954) | BP | 13 | 0.16 | 3 | 18.62 | 0.043 |
| long-term synaptic potentiation (GO:0060291) | BP | 31 | 0.38 | 4 | 10.41 | 0.043 |
| stem cell differentiation (GO:0048863) | BP | 119 | 1.47 | 7 | 4.75 | 0.044 |
| growth plate cartilage development (GO:0003417) | BP | 32 | 0.4 | 4 | 10.09 | 0.045 |
| neuron fate commitment (GO:0048663) | BP | 57 | 0.71 | 5 | 7.08 | 0.046 |
| regulation of blood circulation (GO:1903522) | BP | 121 | 1.5 | 7 | 4.67 | 0.048 |
| positive regulation of neurogenesis (GO:0050769) | BP | 239 | 2.96 | 10 | 3.38 | 0.049 |
| GABA-ergic synapse (GO:0098982) | CC | 39 | 0.48 | 9 | 18.62 | <0.001 |
| dendrite membrane (GO:0032590) | CC | 24 | 0.3 | 5 | 16.81 | <0.001 |
| voltage-gated potassium channel complex (GO:0008076) | CC | 65 | 0.81 | 11 | 13.65 | <0.001 |
| Schaffer collateral - CA1 synapse (GO:0098685) | CC | 39 | 0.48 | 6 | 12.41 | <0.001 |
| glutamatergic synapse (GO:0098978) | CC | 177 | 2.19 | 17 | 7.75 | <0.001 |
| dendrite (GO:0030425) | CC | 263 | 3.26 | 15 | 4.6 | <0.001 |
| extracellular matrix (GO:0031012) | CC | 304 | 3.77 | 15 | 3.98 | <0.001 |
| axon (GO:0030424) | CC | 329 | 4.08 | 16 | 3.92 | <0.001 |
| cell junction (GO:0030054) | CC | 517 | 6.41 | 19 | 2.97 | <0.001 |
| hippocampal mossy fiber to CA3 synapse (GO:0098686) | CC | 17 | 0.21 | 4 | 18.98 | 0.003 |
| GABA receptor complex (GO:1902710) | CC | 22 | 0.27 | 4 | 14.67 | 0.006 |
| integral component of postsynaptic density me…(GO:0099061) | CC | 22 | 0.27 | 4 | 14.67 | 0.006 |
| neuronal cell body membrane (GO:0032809) | CC | 11 | 0.14 | 3 | 22 | 0.012 |
| intrinsic component of presynaptic membrane (GO:0098889) | CC | 32 | 0.4 | 4 | 10.09 | 0.019 |
| excitatory synapse (GO:0060076) | CC | 16 | 0.2 | 3 | 15.13 | 0.029 |
| cell surface (GO:0009986) | CC | 503 | 6.23 | 15 | 2.41 | 0.036 |
| anchored component of membrane (GO:0031225) | CC | 69 | 0.86 | 5 | 5.85 | 0.04 |
| delayed rectifier potassium channel activity (GO:0005251) | MF | 27 | 0.33 | 6 | 17.93 | <0.001 |
| transmitter-gated ion channel activity… (GO:0022824) | MF | 57 | 0.71 | 9 | 12.74 | <0.001 |
| frizzled binding (GO:0005109) | MF | 40 | 0.5 | 6 | 12.1 | 0.002 |
| receptor ligand activity (GO:0048018) | MF | 326 | 4.04 | 15 | 3.71 | 0.002 |
| ligand-gated ion channel activity involved…(GO:0099507) | MF | 3 | 0.04 | 3 | 80.68 | 0.003 |
| G protein-coupled serotonin receptor activity (GO:0004993) | MF | 25 | 0.31 | 5 | 16.14 | 0.003 |
| neuropeptide receptor activity (GO:0008188) | MF | 46 | 0.57 | 6 | 10.52 | 0.003 |
| inhibitory extracellular ligand-gated ion chann…(GO:0005237) | MF | 16 | 0.2 | 4 | 20.17 | 0.007 |
| ligand-gated cation channel activity (GO:0099094) | MF | 107 | 1.33 | 8 | 6.03 | 0.007 |
| cell adhesion molecule binding (GO:0050839) | MF | 142 | 1.76 | 9 | 5.11 | 0.008 |
| channel regulator activity (GO:0016247) | MF | 82 | 1.02 | 7 | 6.89 | 0.008 |
| ligand-gated anion channel activity (GO:0099095) | MF | 18 | 0.22 | 4 | 17.93 | 0.01 |
| excitatory extracellular ligand-gated ion chann…(GO:0005231) | MF | 41 | 0.51 | 5 | 9.84 | 0.016 |
| cell-cell adhesion mediator activity (GO:0098632) | MF | 21 | 0.26 | 4 | 15.37 | 0.016 |
| GABA receptor activity (GO:0016917) | MF | 24 | 0.3 | 4 | 13.45 | 0.023 |
| G protein-coupled acetylcholine receptor activity (GO:0016907) | MF | 9 | 0.11 | 3 | 26.89 | 0.024 |
| chloride channel activity (GO:0005254) | MF | 51 | 0.63 | 5 | 7.91 | 0.037 |

**Supplementary Table S8**. Genes under positive selection, including their relative expression (low = TPM≤10; medium = 10>TPM<50; high = TPM≥50), the tissue with the highest expression (TPM), and their index of tissue specificity (τ). ‘Symbol’ denotes human orthologs.

| **Symbol** | **Description** | **relative**  **expression** | **Tissue with**  **max expression** | **τ** |
| --- | --- | --- | --- | --- |
| CNRIP1 | CB1 cannabinoid receptor-interacting protein 1 | High | brain | 0.83 |
| ICOS | inducible T-cell costimulator | High | spleen | 0.81 |
| PTTG1 | securin | High | ovary | 0.63 |
| GTF2IRD1 | general transcription factor II-I repeat domain-containing protein 1 isoform X6 | High | brain | 0.59 |
| HEMGN | hemogen | High | blood | 0.59 |
| CDCA9 | borealin-2-like | High | ovary | 0.57 |
| LACC1 | laccase domain-containing protein 1 isoform X1 | High | spleen | 0.56 |
| EIF4E3 | eukaryotic translation initiation factor 4E type 3 | High | brain | 0.55 |
| PJA2 | E3 ubiquitin-protein ligase Praja-2 isoform X1 | High | ovary | 0.54 |
| CCDC40 | coiled-coil domain-containing protein 40 | High | spleen | 0.52 |
| MTUS1 | microtubule-associated tumor suppressor 1 isoform X1 | High | ovary | 0.51 |
| AK7 | adenylate kinase 7 isoform X1 | High | ovary | 0.47 |
| TRIM25 | E3 ubiquitin/ISG15 ligase TRIM25 | High | ovary | 0.46 |
| TSPAN2 | tetraspanin-2 | High | spleen | 0.46 |
| NTPCR | cancer-related nucleoside-triphosphatase isoform X1 | High | ovary | 0.43 |
| CIPC | CLOCK-interacting pacemaker | High | ovary | 0.41 |
| SUFU | suppressor of fused homolog isoform X2 | High | ovary | 0.4 |
| LIPT1 | lipoyltransferase 1, mitochondrial | High | ovary | 0.38 |
| EIF2B4 | translation initiation factor eIF-2B subunit delta isoform X2 | High | ovary | 0.37 |
| TMEM170A | transmembrane protein 170A | High | brain | 0.37 |
| WDSUB1 | WD repeat, SAM and U-box domain-containing protein 1 isoform X4 | High | brain | 0.37 |
| CCDC34 | coiled-coil domain-containing protein 34 | High | ovary | 0.36 |
| TMEM206 | transmembrane protein 206 | High | ovary | 0.31 |
| DBF4 | protein DBF4 homolog A isoform X2 | High | spleen | 0.29 |
| CPPED1 | serine/threonine-protein phosphatase CPPED1 isoform X1 | High | brain | 0.27 |
| SNAPC4 | snRNA-activating protein complex subunit 4 | High | spleen | 0.27 |
| USP14 | ubiquitin carboxyl-terminal hydrolase 14 isoform X2 | High | ovary | 0.27 |
| DIABLO | diablo homolog, mitochondrial | High | ovary | 0.24 |
| ALKBH5 | RNA demethylase ALKBH5 | High | ovary | 0.23 |
| NARS2 | probable asparagine--tRNA ligase, mitochondrial | High | ovary | 0.22 |
| RMDN3 | regulator of microtubule dynamics protein 3 isoform X3 | High | brain | 0.22 |
| RPS11 | 40S ribosomal protein S11 | High | spleen | 0.21 |
| MKKS | McKusick-Kaufman/Bardet-Biedl syndromes putative chaperonin | High | spleen | 0.19 |
| DNTTIP2 | deoxynucleotidyltransferase terminal-interacting protein 2 | High | spleen | 0.14 |
| OCIAD1 | OCIA domain-containing protein 1 | High | ovary | 0.1 |
| IL12B | interleukin-12 subunit beta | Medium | ovary | 0.76 |
| DEUP1 | deuterosome protein 1 isoform X2 | Medium | brain | 0.68 |
| FAM189A1 | protein FAM189A1 isoform X1 | Medium | brain | 0.66 |
| EDAR | tumor necrosis factor receptor superfamily member EDAR isoform X1 | Medium | spleen | 0.64 |
| RMI1 | recQ-mediated genome instability protein 1 | Medium | ovary | 0.55 |
| TMEM116 | transmembrane protein 116 | Medium | ovary | 0.5 |
| DRC7 | dynein regulatory complex subunit 7 | Medium | ovary | 0.49 |
| DECR2 | peroxisomal 2,4-dienoyl-CoA reductase isoform X2 | Medium | brain | 0.47 |
| FAM161A | protein FAM161A | Medium | spleen | 0.33 |
| MAB21L4 | uncharacterized protein C2orf54 homolog | Low | ovary | 0.38 |
| AKAP14 | A-kinase anchor protein 14 | Low | brain | 0.36 |

**Supplementary Table S9**. Sequence information for the coding sequences of the 8 bird species obtained from NCBI and the newly sequenced transcriptome of the tree swallow (*Tachycineta bicolor*).

| **Species** | **# Peptides** | **# Peptides post filter** | **# AA after filter** | **Genome version** | **Protein/GFF source** | **CDS source** |
| --- | --- | --- | --- | --- | --- | --- |
| *Zonotrichia albicollis* | 23892 | 14379 | 8415689 | GCF_000385455.1_Zonotrichia_albicollis-1.0.1 | ftp://ftp.ncbi.nlm.nih.gov/genomes/Zonotrichia_albicollis/ | ftp://ftp.ncbi.nlm.nih.gov/genomes/all/GCF/000/385/455/GCF_000385455.1_Zonotrichia_albicollis-1.0.1/ |
| *Corvus brachyrhynchos* | 29421 | 14927 | 8678392 | GCF_000691975.1_ASM69197v1 | ftp://ftp.ncbi.nlm.nih.gov/genomes/Corvus_brachyrhynchos/ | ftp://ftp.ncbi.nlm.nih.gov/genomes/all/GCF/000/691/975/GCF_000691975.1_ASM69197v1/ |
| *Parus major* | 39666 | 15240 | 9143512 | GCF_001522545.2_Parus_major1.1 | ftp://ftp.ncbi.nlm.nih.gov/genomes/Parus_major/ | ftp://ftp.ncbi.nlm.nih.gov/genomes/all/GCF/001/522/545/GCF_001522545.2_Parus_major1.1/ |
| *Ficedula albicollis* | 26464 | 15387 | 9062363 | GCF_000247815.1_FicAlb1.5 | ftp://ftp.ncbi.nlm.nih.gov/genomes/Ficedula_albicollis/ | ftp://ftp.ncbi.nlm.nih.gov/genomes/all/GCF/000/247/815/GCF_000247815.1_FicAlb1.5/ |
| *Taeniopygia guttata* | 19443 | 16355 | 8834332 | GCF_000151805.1_Taeniopygia_guttata-3.2.4 | ftp://ftp.ncbi.nlm.nih.gov/genomes/Taeniopygia_guttata/ | ftp://ftp.ncbi.nlm.nih.gov/genomes/all/GCF/000/151/805/GCF_000151805.1_Taeniopygia_guttata-3.2.4/ |
| *Cyanistes caeruleus* | 31326 | 16519 | 8971750 | GCF_002901205.1_cyaCae2 | ftp://ftp.ncbi.nlm.nih.gov/genomes/Cyanistes_caeruleus/ | ftp://ftp.ncbi.nlm.nih.gov/genomes/all/GCF/002/901/205/GCF_002901205.1_cyaCae2/ |
| *Sturnus vulgaris* | 26611 | 15233 | 9084104 | GCF_001447265.1_Sturnus_vulgaris-1.0 | ftp://ftp.ncbi.nlm.nih.gov/genomes/Sturnus_vulgaris/ | ftp://ftp.ncbi.nlm.nih.gov/genomes/all/GCF/001/447/265/GCF_001447265.1_Sturnus_vulgaris-1.0/ |
| *Gallus gallus* | 49673 | 17477 | 10095268 | GCF_000002315.5_GRCg6a | ftp://ftp.ncbi.nlm.nih.gov/genomes/Gallus_gallus/ | ftp://ftp.ncbi.nlm.nih.gov/genomes/all/GCF/000/002/315/GCF_000002315.5_GRCg6a/ |
| *Tachycineta bicolor* | NA | 14717 | 7875811 | NA | NA | NA |


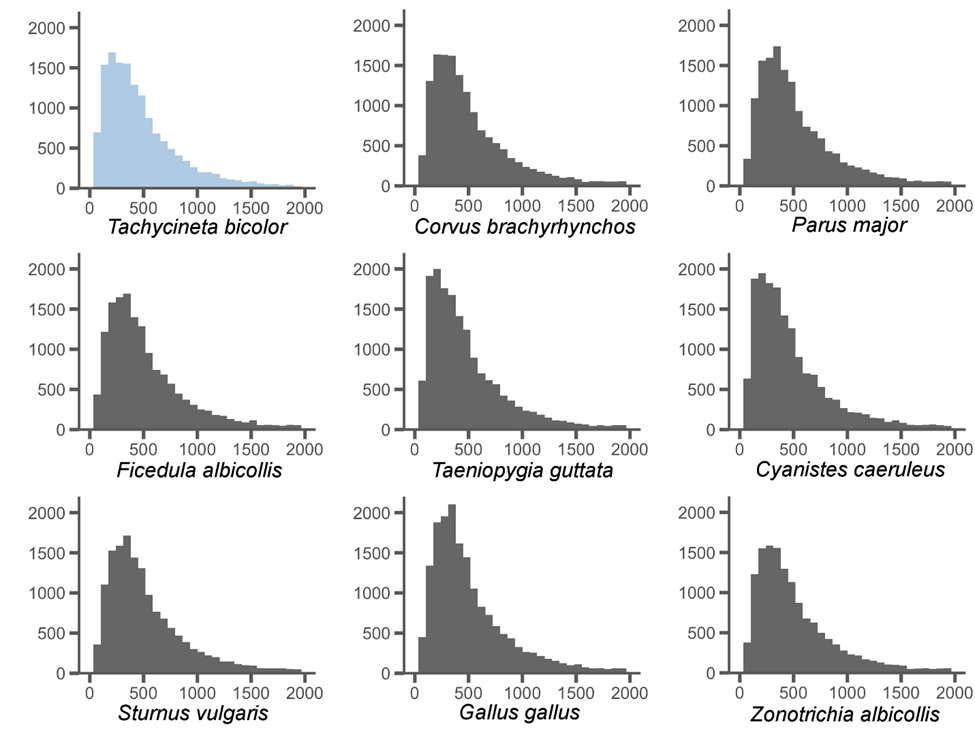


**Supplementary Figure S1**. Sequence length distributions for the 8 species included in the positive selection analysis (grey) and the *de novo* assembly of the tree swallow transcriptome (all tissues combined; light blue).


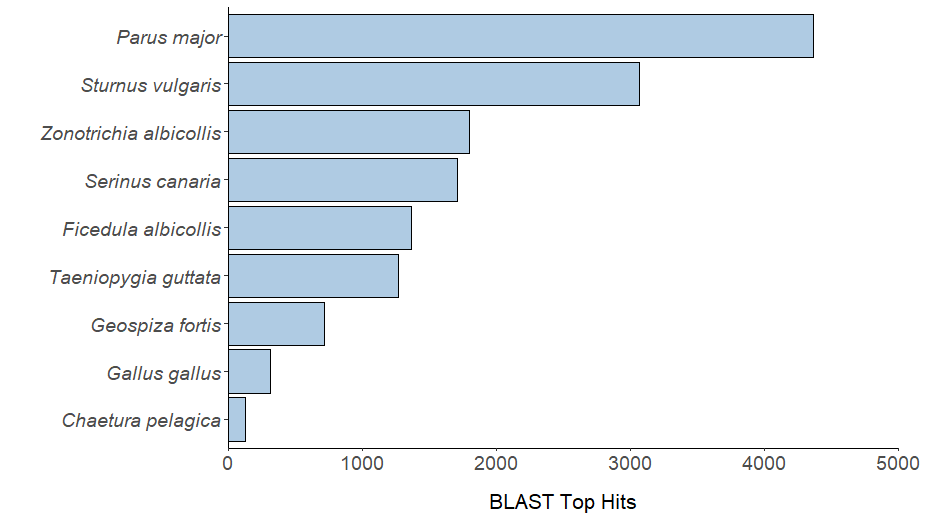


**Supplementary Figure S2**. Species distribution of the top BLAST hits for the annotation of the *de novo* assembly of the tree swallow transcriptome (n=14,717 protein-coding genes).


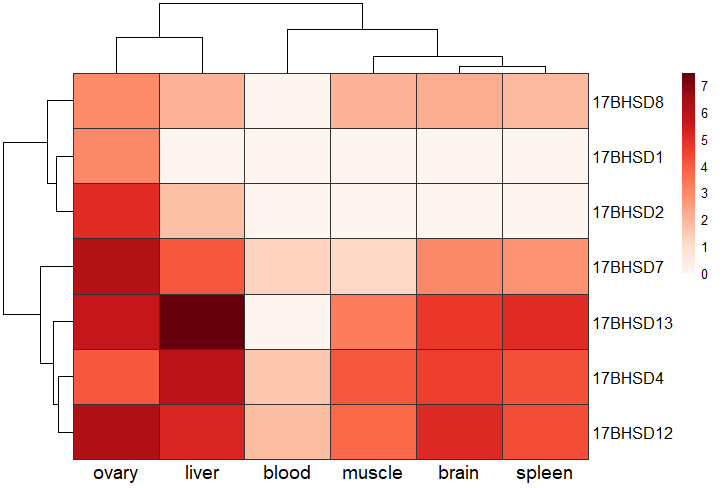


**Supplementary Figure S3**. Expression of 17βHSD isoforms across all tissues sampled in this study. Scale depicts log2 expression and isoforms are clustered by rows using Euclidean distance.


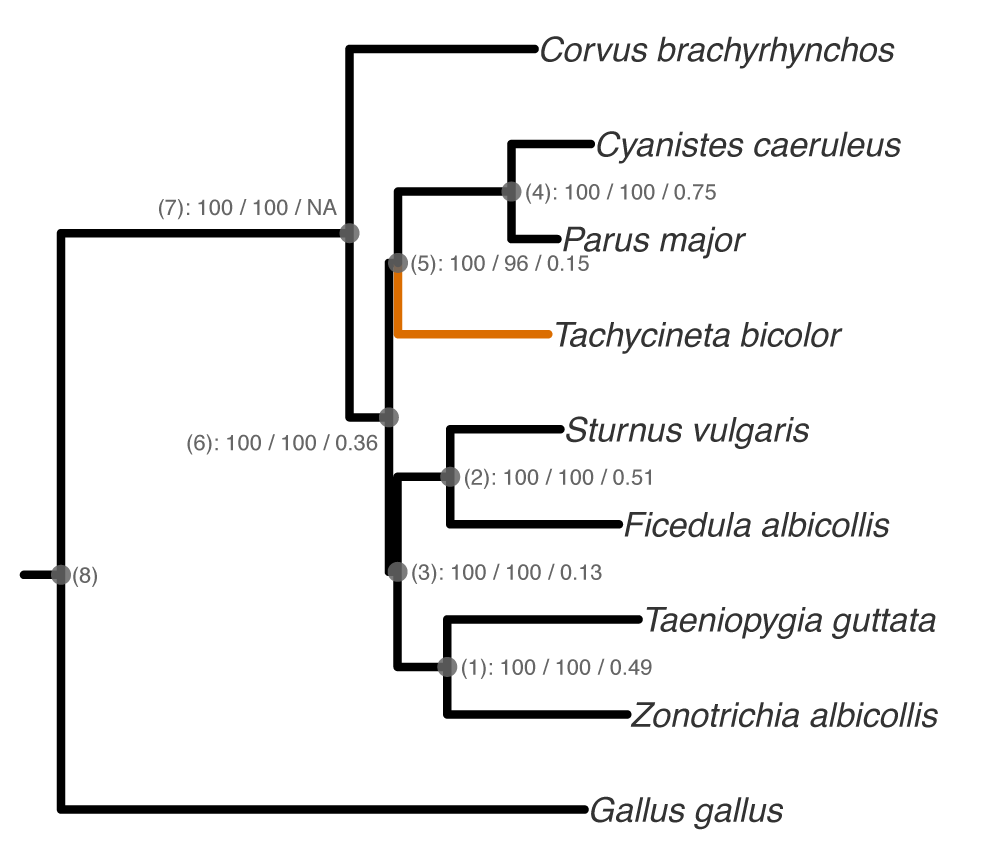


**Supplementary Figure S4.** Nine species phylogeny generated in the positive selection analysis. Branch lengths inferred from concatenated alignment of 3,015 coding sequences. Nodes labeled as: (Node ID): ASTRAL bootstrap / Concatenation bootstrap / Concordance factor. Bootstraps provides a measure of confidence in sampling variance while concordance factors better summarize underlying phylogenetic signal.

**Supplementary Methods: Positive Selection Analysis**

We constructed species and gene phylogenies and assessed positive selection in the tree swallow transcriptome by comparing it to 8 other bird species: white-throated sparrow (*Zonotrichia albicollis*), American crow (*Corvus brachyrhynchos*), great tit (*Parus major*), collared flycatcher (*Ficedula albicolllis*), zebra finch (*Taeniopygia guttata*), blue tit (*Cyanistes caeruleus*), starling (*Sturnus vulgaris*), and chicken (*Gallus gallus*). We downloaded the peptides and coding nucleotide sequences for these 8 species from the NCBI database on July 27, 2018 and filtered them such that only the longest isoform of each gene was retained. To start clustering the peptides into orthologous groups for analysis we performed an all-v-all BLAST^1^ search on the filtered protein sequences from these 8 species plus the 14,717 predicted peptides for the tree swallow (see Supplementary Table S7). The resulting e-values from the search were used as the main clustering criterion for the MCL program to place the peptides into 10,641 orthologous groups^2^.

*Phylogeny reconstruction & incomplete lineage sorting (ILS) in the bird phylogeny*

To reconstruct the phylogeny of these bird species, we used the 3,015 single-copy peptide groups that have exactly one copy of the gene present in each species. We aligned these groups with PASTA^3^ and then reconstructed the bird phylogeny in two ways. First, we reconstructed gene trees for each locus individually with RAxML^4^ with the PROTGAMMAJTTF amino acid model and then used those gene trees as the input for the quartet summary method ASTRAL^5^ to infer a species tree. We also concatenated all 3,015 alignments into a single alignment file and again used RAxML (PROTGAMMAJTTF amino acid model) to infer the species tree from this concatenated alignment alone. Both reconstruction methods give the same tree topology for these 9 species and both also result in trees that are almost completely supported by bootstrap resampling (Supplementary Figure S4). However, bootstrap support may be misleading because it does not actually capture incongruence in the resulting gene trees. To assess incongruence, we measured concordance factors of the gene trees by rooting them with *Gallus gallus* as the outgroup and comparing them to the ASTRAL species tree. It should be noted that the rooting procedure artificially forces the node leading to the ingroups (node 7 in Supplementary Fig. S4) to have a concordance factor of 1, and as such it is excluded from consideration. We define the concordance factor of a node in the species tree (
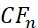
) as the number of times that node appears in a gene tree divided by the total number of gene trees:


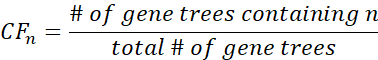


We observed a discrepancy between concordance factors and bootstrap support for the ASTRAL species tree. Notably, no node has a concordance factor above 0.75, meaning no node appears in more than 75% of the gene trees. Of particular interest are the nodes 5 and 3 which have concordance factors of 0.15 and 0.13, respectively (See Supplementary Fig. S4). Branch lengths inferred from the concatenated alignment for the lineages leading to these two nodes are extremely short. These low concordance factors and short branch lengths point to the possibility of high amounts of ILS in the ancestors of these clades. This can lead to the observation of unsorted ancestral polymorphism which can in turn mislead tests that use the species tree to calculate values related to sequence divergence^6^. We address this issue by using individual gene trees rather than a single species tree in our tests for positive selection.

**Newick formatted species trees:**

*ASTRAL (nodes labeled as <Node ID>_concordance factor_bootstrap support):*

(Gallus_gallus,((((Zonotrichia_albicollis,Taeniopygia_guttata)<1>_0.49_100.0,(Ficedula_albicollis,Sturnus_vulgaris)<2>_0.51_100.0)<3>_0.13_100.0,(Tachycineta_bicolor,(Parus_major,Cyanistes_caeruleus)<4>_0.75_100.0)<5>_0.15_96.0)<6>_0.36_100.0,Corvus_brachyrhynchos)<7>_1.0)<8>_1.0

*Concatenation (branches labeled as relative number of substitutions; nodes labeled as <Node ID>_bootstrap support):*

(Gallus_gallus:0.11745,(Corvus_brachyrhynchos:0.04152,(((Zonotrichia_albicollis:0.04037,Taeniopygia_guttata:0.04292)<1>_100:0.01114,(Sturnus_vulgaris:0.02472,Ficedula_albicollis:0.03784)<2>_100:0.01180)<3>_100:0.00196,((Parus_major:0.01031,Cyanistes_caeruleus:0.01780)<4>_100:0.02545,Tachycineta_bicolor:0.03370)<5>_100:0.00205)<6>_100:0.00883)<7>:0.06473)<8>;

*Inferring positive selection on the tree swallow lineage*

To determine the genes evolving under positive selection along the tree swallow lineage (orange branch in Supplementary Fig. S4), we set that branch of the phylogeny as the foreground branch in PAML’s^7^ branch-site test^8^. This test compares two likelihood models of sequence divergence, one in which the ratio of non-synonymous to synonymous substitutions (*ω*) is restricted to a value of one (model=2, NSsite=2, omega=1, fix_omega=1) on the foreground branch to a model in which *ω* is not restricted and is free to take values above 0 (model=2, NSsite=2, omega=1, fix_omega=0). Genes that show significance in a likelihood ratio test of these two models with a conservative test statistic value greater than 5.99^8^ are then said to be evolving under positive selection.

To prepare our data for the branch-site test, we aligned the coding sequences from the 3,015 single-copy genes with two codon alignment programs: PRANK^9^ and MACSE^10^. By replicating the analysis with two alignment programs we can look for overlapping genes in the final lists of genes inferred as evolving under positive selection by PAML and eliminate possible errors due to poor alignments. Prior to running them through PAML, alignments were also masked with GBlocks^11^ using default parameters to remove poorly aligned or gap-ridden positions. Finally, because we observed ILS in our phylogeny, we ran the branch-site test on the coding sequence of each gene using the gene tree inferred from the coding sequence of that locus, rather than using the inferred species tree for all loci. This helps minimize the effect of substitutions produced by ILS^6^. Gene trees for the coding sequences were also inferred with RAxML (GTRGAMMA nucleotide model). Running the branch-site test on our set of coding sequences yields 76 genes with significant evidence for positive selection in the tree swallow in the MACSE dataset, 58 genes in the PRANK dataset, and 46 genes overlapping between the two sets. We used the set of 46 overlapping genes to draw conclusions about adaptation in the tree swallow in the main text.

**References**

1. Altschul, S. F. *et al.* Gapped BLAST and PSI-BLAST: a new generation of protein database search programs. *Nucleic Acids Res.* **25**, 3389–3402 (1997).

2. Enright, A. J., Van Dongen, S. & Ouzounis, C. A. An efficient algorithm for large-scale detection of protein families. *Nucleic Acids Res.* **30**, 1575–1584 (2002).

3. Mirarab, S. *et al.* PASTA: ultra-large multiple sequence alignment for nucleotide and amino-acid sequences. *J. Comput. Biol.* **22**, 377–386 (2015).

4. Stamatakis, A. RAxML version 8: a tool for phylogenetic analysis and post-analysis of large phylogenies. *Bioinformatics* **30**, 1312–1313 (2014).

5. Sayyari, E. & Mirarab, S. Fast coalescent-based computation of local branch support from quartet frequencies. *Mol. Biol. Evol.* **33**, 1654–1668 (2016).

6. Mendes, F. K. & Hahn, M. W. Gene tree discordance causes apparent substitution rate variation. *Syst. Biol.* **65**, 711–721 (2016).

7. Yang, Z. PAML 4: phylogenetic analysis by maximum likelihood. *Mol. Biol. Evol.* **24**, 1586–1591 (2007).

8. Zhang, J., Nielsen, R. & Yang, Z. Evaluation of an improved branch-site likelihood method for detecting positive selection at the molecular level. *Mol. Biol. Evol.* **22**, 2472–2479 (2005).

9. Löytynoja, A. Phylogeny-aware alignment with PRANK. In *Multiple Sequence Alignment Methods* (Russell, D. J.) 155–170 (Springer, 2014).

10. Ranwez, V. *et al.* MACSE v2: toolkit for the alignment of coding sequences accounting for frameshifts and stop codons. *Mol. Biol. Evol.* **35**, 2582–2584 (2018).

11. Castresana, J. Selection of conserved blocks from multiple alignments for their use in phylogenetic analysis. *Mol. Biol. Evol.* **17**, 540–552 (2000).
